# Prognostic Value of Frailty for Older Patients with Heart Failure: A Systematic Review and Meta-Analysis of Prospective Studies

**DOI:** 10.1155/2018/8739058

**Published:** 2018-10-22

**Authors:** Xige Wang, Changli Zhou, Yuewei Li, Huimin Li, Qinqin Cao, Feng Li

**Affiliations:** School of Nursing, Jilin University, 965 Xinjiang Street, Changchun, Jilin 130020, China

## Abstract

**Objective:**

Numerous studies have investigated the prognostic role of frailty in elderly patients with heart failure (HF), but the limited size of the reported studies has resulted in continued uncertainty regarding its prognostic impact. The aim of this study was to integrate the findings of all available studies and estimate the impact of frailty on the prognosis of HF by performing a systematic review and meta-analysis.

**Methods:**

PubMed, Embase, Cochrane, and Web of Science databases were searched from inception to November 8^th^ 2017 to identify eligible prospective studies. The Newcastle-Ottawa Scale (NOS) was used to evaluate study quality. The association between frailty and HF outcomes was reviewed. Overall hazard ratios (HRs) for the effects of frailty on all-cause mortality were pooled using a fixed-effect model and publication bias was evaluated using funnel plots.

**Results:**

A total of 10 studies involving 3033 elderly patients with HF were included in the systematic review and meta-analysis. All eligible studies indicated that frailty was of prognostic significance for HF patients. The HRs for the effects of frailty on all-cause mortality were 1.70 (95% confidence interval (CI): 1.41–2.04), based on the pooling of six studies that provided related data. However, publication bias was observed among the studies.

**Conclusions:**

Frailty has a high prevalence among older patients with HF. Elderly HF patients with frailty have a poorer prognosis than those without frailty. Further studies are now required to implement the use of frailty assessment tools and explore effective interventions for frailty in older HF patients.

## 1. Introduction

Due to the increased age of the general population, HF patients are predominantly elderly [1, 2]. HF is the most common cause of hospitalization in the older population leading to a high risk of mortality, disability, and hospital readmission [3, 4]. Due to poor prognosis and high costs of treatment, adequate risk assessments and optimized treatment decisions are essential in older patients with HF [5]. In the past, the assessment of care for HF patients was primarily based on biological age and subjective symptoms. However, the health trajectory of the elderly is complex and cannot simply be interpreted by physical age criteria or a single disease-specific risk factor [6]. These indicators do not reflect the actual physical health status of older patients, and so current assessments do not fully explain the risk of HF in the elderly [7, 8].

Similar to HF, frailty is a geriatric syndrome characterised by decreased physiological activity of multiple organ systems as a result of aging. Frail elderly individuals are prone to adverse events, including falls and hospitalization [9–11]. In recent years, as research on frailty has increased, a high incidence has been identified in HF patients, increasing the risk of adverse outcomes [12, 13]. This may reflect the central role of the heart in the perfusion of all organs throughout the body and the diffuse damage caused by HF. It may also reflect the common pathophysiological links between HF and frailty [14, 15].

Despite numerous studies showing an adverse prognostic impact of frailty on clinical outcomes including survival, the limited size of the individual studies has resulted in persisting uncertainty regarding the prognostic impact of frailty in the older HF population. We therefore performed a systematic review and meta-analysis to integrate the findings of available studies and estimate the prognostic value of frailty for older patients with HF.

## 2. Methods

We performed a systematic review and meta-analysis of prospective studies to estimate the prognostic value of frailty for older patients with HF. This systematic review and meta-analysis was performed according to PRISMA guidelines.

### 2.1. Eligibility Criteria

Studies were required to meet all of the following inclusion criteria: (1) patients: subjects with HF, age ≥ 65 years; (2) interventions: subjects with frailty; (3) comparators: subjects without frailty; (4) outcomes: all-cause mortality or all-cause hospitalization or HF-related hospitalization. Hazard ratios (HRs) with 95% confidence intervals (CI) had to be included.

### 2.2. Information Sources

PubMed, Embase, Cochrane, and the Web of Science databases, from inception to November 8th 2017, were searched for relevant articles.

### 2.3. Search Criteria

The search strategy included Mesh terms for “Frail”, “Elderly”, and “Heart Failure”. The language used in included manuscripts was limited to English.

### 2.4. Study Selection

Abstracts of retrieved articles were independently screened by two investigators (Wang and Zhou). Articles that did not meet the inclusion criteria were excluded and the remaining articles were fully reviewed. Any disagreements were resolved by the third reviewer (Cao).

### 2.5. Data Items and Data Collection Process

A standardized data collection form was used to extract the following information: first author's name, study country, year of publication, number of participants, mean age of sample, proportion of women in the study population, HF type, proportion of HF reduced Ejection Fraction (HFrEF) or HF preserved Ejection Fraction (HFpEF) patients, mean Ejection Fraction (EF), proportion of New York Heart Association (NYHA) class III-IV patients, definition of frailty, duration of follow-up, method of frailty measurement, prevalence of frailty in the sample, endpoints with corresponding HRs and 95% CIs, and confounding factors adjusted for. We adopted the adjusted HR if both adjusted and crude estimates were provided. The data extraction process was independently performed by two investigators (Wang and Zhou). Any disagreements were resolved by the third reviewer (Cao).

### 2.6. Risk of Bias in Individual Studies

Two reviewers (Wang and Zhou) independently assessed the quality of included articles using the Newcastle-Ottawa Scale (NOS) [16]. The quality assessment tool evaluates the bias risk of each study from three aspects: (1) selection of the exposed and unexposed study populations; (2) comparability between the two groups; (3) outcome measurements. The maximum of these three dimensions is 4, 2, and 3, respectively. Any disagreements were resolved by the third reviewer (Cao).

### 2.7. Statistical Analysis

Review Manager 5.3 software was used for all data analysis. Only studies that used validated methods to assess frailty and provided HRs and 95% CIs for the mortality of older HF patients were included in the meta-analysis. Statistical heterogeneity among studies was assessed by Cochran's Q test and I^2^ statistics, which indicate the percentage of total variation between studies due to heterogeneity rather than chance. If a low heterogeneity was observed (I^2^ < 50% or P> 0.10), we pooled the reported HRs using a fixed-effect model with the generic inverse variance method [17]. Otherwise, all HRs were pooled using a random-effects model via the same method. Potential publication bias was evaluated using the funnel plot [18].

## 3. Results

### 3.1. Study Selection

**Figure 1:**
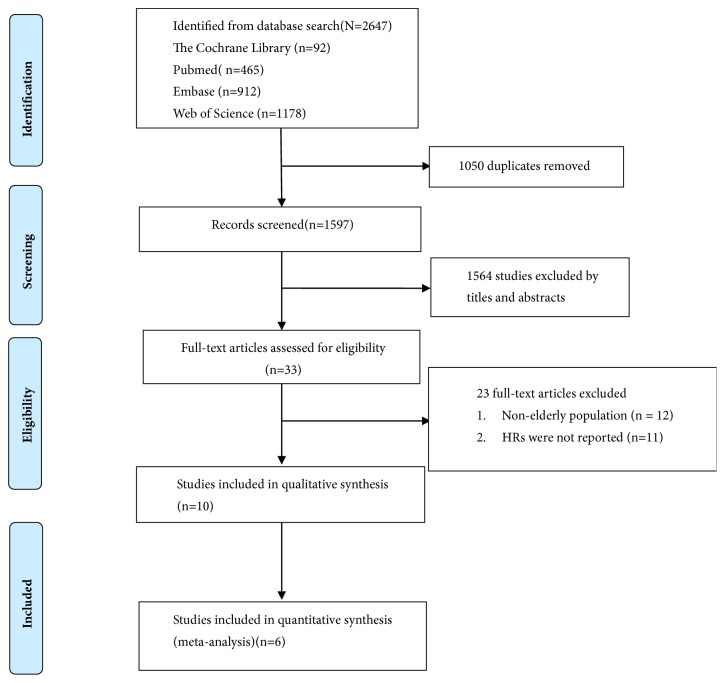
Literature screening process.

The flow chart of the literature retrieval is shown in Figure 1. After reviewing the full-text articles, 23 studies were excluded as they included nonelderly subjects (n = 12) or did not report HR (n=11). Ultimately, 10 prospective cohort studies were included in the systematic review [19–28].

### 3.2. Study Characteristics

Table 1 summarizes the characteristics of the ten studies included in the systematic review. Four studies were conducted in Italy [20, 22, 26, 28], three in the USA [19, 25, 27], and three in Spain [21, 23, 24]. In total, the studies included 3033 elderly patients with HF. The sample sizes of the included articles were relatively small with 2 of the studies including less than 100 participants [19, 27]. Nine studies reported a mean age ranging from 74.9 to 85.2 years. One study failed to report the mean age. The majority of articles had almost equal male: female ratios, with only one article reporting a smaller number of women (28.81%) [19]. In the included studies, the shortest follow-up period was 30 days and the longest was 20 years. The prevalence of frailty in older HF patients ranged from 25.4% to 76%.

### 3.3. Frailty Assessment

Eight studies were classified as “Physical Frailty” as they primarily used physical frailty assessment tools such as the Fried Frailty Phenotype (FP) measurement [19, 21, 23, 24, 27], portions of the five FP indicators (e.g., gait speed or handgrip strength)[25, 28], or the Short Physical Performance Battery (SPPB) [26]. Two studies were classified as “Multidimensional Frailty” as they used a multidimensional frailty measurement tool: Frailty Staging System (FSS) [20, 22], which evaluated multiple domains of elderly functioning.

### 3.4. Main Findings of the Eligible Studies

Table 2 highlights the main study findings. The association between frailty status and all-cause mortality was investigated in eight studies [19–24, 26, 28]. Three studies investigated the impact of frailty status on all-cause hospitalization [25, 27, 28] and two studies investigated the effect of frailty on HF-related hospitalization [27, 28]. The effects of frailty on readmission [23], 1-year readmission [24], 1-year readmission for HF [22], all-cause hospitalization or death [27], incidental functional limitations [23], and 30-day functional decline [24] were also investigated in some of the studies.

Detailed confounding factors adjusted for estimates of HRs are shown in Table 2. Well-characterized confounding factors include age (n =9), sex (n= 7), NYHA class (n =7), EF (n= 6), beta-blocker therapy (n= 4), and systolic blood pressure (n= 3).

### 3.5. Association between Frailty and Mortality in HF Patients

**Figure 2:**
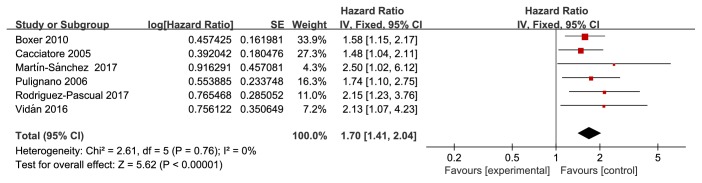
Forest plot of the HR for frailty for all-cause mortality in older HF patients.

Eight studies reported the adjusted HRs for frailty for all-cause mortality in older HF patients [19–24, 26, 28]. Two studies were excluded due to the assessment tool for frailty and comparison of mortality rates among different frailty-status groups, leaving six articles that were eventually included in the meta-analysis [19–24].  Figure 2 depicts the summary effect estimated using the fixed-effects model. The pooled HR showed that, for older patients with HF, frailty was associated with a 70% higher risk of future mortality events (summary HR: 1.70, 95% CI: 1.41–2.04). I^2^ = 0 (P<0.00001), suggesting low statistical heterogeneity among the studies included in the meta-analysis.

### 3.6. Methodological Quality Assessment

The quality assessment of the included studies is described in Table 2. For quality assessments, four studies were considered as having mild cohort selection bias as they were designed as multicenter studies [21, 23, 26, 28]. One study was regarded as having high outcome bias, as it did not describe the outcome of the loss or evaluation methods of the endpoints [22]. One study was regarded as having mild outcome bias due to the short follow-up period [21].

### 3.7. Evaluation for Publication Bias

**Figure 3:**
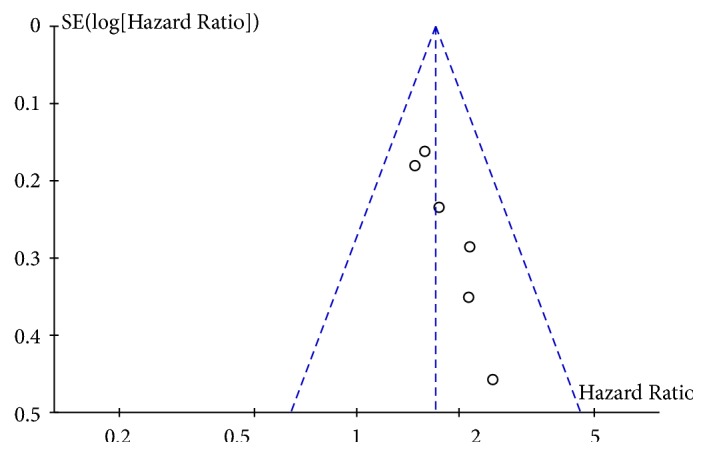
Funnel plot.

Obvious asymmetrical pattern is observed in Figure 3, indicating that potential publication bias existed across the studies.

## 4. Discussion

### 4.1. Frailty in Older HF Patients

This study showed that the prevalence of frailty in older patients with HF ranged from 25.4% to 76%, similar to the 15% to 74% range in HF patients described in previous studies [29]. According to Fried's definition, the prevalence of frailty in the elderly community is 14% [9]. Thus, this study confirmed that the prevalence of frailty in older patients with HF is significantly higher than normal elderly population. Frailty was also an independent risk factor for HF in elderly patients. Compared to nonfrail older adults, the risk of HF in severely frail elderly patients increased by 88% [30]. Thus, it can be considered that HF and frailty are interrelated. HF increases the likelihood of becoming frail and frailty increases the risk of HF.

In addition, fatigue, weakness, and activity intolerance are common manifestations of HF and frailty [31, 32]. The underlying mechanisms of clinical similarities and coexistence may be the common pathological pathways involved in the two syndromes, including inflammatory processes and endocrine, metabolic, and autonomic dysfunction. The inflammatory process is the most critical of the aforementioned pathways [33, 34]. Inflammation is known to play an important role in the development of cardiovascular diseases, including HF. Tissue depletion and cardiac cachexia are associated with elevated interleukin-1 (IL-1), interleukin-6 (IL-6), C-reactive protein (CRP), and tumor necrosis factor-*α* (TNF-*α*) [32]. In addition, studies have found that inflammation is related to an increased incidence of frailty, and the levels of IL-6, CRP, TNF-*α*, and other inflammatory markers among frail and elderly subjects were elevated [33, 34]. Elevated levels of inflammatory markers, particularly TNF-*α* and its soluble receptors, may lead to decreased muscle mass and strength by promoting catabolic processes in muscle cells. The loss of muscle mass is an important component of frailty. Results from Women's Health and Aging studies revealed an increased risk of frailty with an increased number of concurrent inflammatory diseases, further validating the inflammatory nature of frailty [33].

The exact mechanisms of frailty in older patients with HF remain unclear. It is likely that common pathophysiological processes between the two diseases reinforce each other through complex cellular and molecular mechanisms.

### 4.2. Frailty and Prognosis in Older HF Patients

We found that frailty was associated with increased adverse outcomes such as hospitalizations and mortality in older patients with HF. In addition, frailty also leads to a decline in the quality of life of older patients with HF [35, 36]. Above all, it was evident that, as a reduced physical reserve and vulnerable geriatric syndrome, frailty has an important effect on the development and prognosis of older patients with HF. Thus, greater attention to the prognostic value of frailty in older patients with HF is required.

Similar to geriatrics, care for patients with HF should broaden the coverage of traditional disease-oriented models. Despite an emphasis on the integrated management of HF patients, the most commonly used tools for assessing functional capacity in HF patients are limited to those that assess patient disability including NYHA, cardiopulmonary exercise testing, and six-minute walk testing [7]. The above assessments do not reflect the prognostic value of frailty in older patients with HF. In addition, frailty had a higher prognostic value for HF patients compared to subjective NYHA assessments. It is worth noting that a study has shown that a frailty assessment is better than traditional HF indexes for predicting short-term (30 days) mortality of older HF patients, suggesting that frailty as a state of systemic metabolic disorders is more important than traditional specificity indicators of HF [37]. Comprehensive and accurate risk assessments will be beneficial for individualized treatment regimens and information shared decision-making. According to our results, frailty plays an important role in the management of older patients with HF. Because frailty is reversible, its early detection in patients with HF helps to actively implement effective interventions, including physical activity and nutritional supplements that improve frailty and HF prognosis.

Furthermore, for patients with advanced HF, cardiac surgery such as heart transplantation and left ventricular assist device implantation often leads to severe postoperative morbidity and mortality during disease treatment. Therefore, the risk stratification should be performed prior to operation by comprehensively evaluating the patient's tolerance and ability to benefit from surgery [31, 33]. At present, the risk assessment system of cardiac surgery is based primarily on chronological age. However, chronological age did not fully reflect the true functional status of the elderly, which can result in an inaccuracy of risk assessments. Recently, related studies have shown that frailty can increase the risk of adverse outcomes following cardiac surgery in the elderly [38, 39]. Thus, frailty may also represent an important tool for stratifying the risk of invasive treatments in patients with HF. In future cardiac procedures, frailty assessments must be incorporated into risk prediction models.

### 4.3. Frailty Assessment in Older HF Patients

Frailty is more prevalent in older patients with HF, and the presence of frailty often suggests a poorer prognosis. Therefore, the early identification of frailty in older patients with HF is crucial. Currently, there is no recognized gold standard for frailty assessment and no frailty assessment tool has been specifically validated in the HF population [40]. The results of this study indicated that frailty assessment tools in older patients with HF are inconsistent and the assessment of physical frailty overlaps with, but is not identical to, frailty instruments that assess multidimensional frailty. In addition, it should be noted that although some studies used validated assessment tools, the assessment process was not rigorous. For example, in studies by Martín-Sánchez and colleagues [21], despite the use of the FP to assess frailty, indicators such as gait speed and handgrip strength were self-reported questions that greatly reduced the accuracy of the assessments. Two articles used FSS to evaluate frailty, but the frailty ratings differed [20, 22].

In summary, the frailty assessment of older patients with HF requires standardization and unification. Otherwise, the differences caused by measurement methods cannot truly reflect the nature of the patient's disease.

### 4.4. Limitations

The present systematic review and meta-analysis has some limitations: (1) Due to the different tools used to evaluate frailty, clinical heterogeneity was unavoidable. (2) The confounding factors adjusted in individual studies varied and some important indicators were not adjusted in some studies, including NYHA class. An inadequate adjustment of confounding factors may overestimate or underestimate the prognostic value of frailty in older HF patients. (3) Only six studies were included in the final meta-analysis and publication bias was observed. Therefore, the prognostic value of frailty for older HF patients may be overestimated. (4) Our study was not registered prior to implementation meaning publication bias may exist. The operational study steps were however strictly in accordance with PRISMA guidelines. (5) HF itself is a heterogeneous disease and the types of HF differed across the studies included in the meta-analysis. Therefore, it is worth noting that the heterogeneity of HF across the study subjects may have affected our estimates of the prognostic value of frailty in older patients with HF. (6) During the selection of included articles, only those published in English were included. Therefore, the study contains selection bias.

## 5. Conclusions

The results of the present systematic review and meta-analysis indicate that frailty is more prevalent in older patients with HF and that frailty increases the risk of death by 70%. Therefore, frailty is an effective indicator of the prognostic evaluation of older HF patients and clinical medical staff should attach importance to the role of frailty assessments during HF management. However, it should also be noted that a lack of standardization and unification of the assessment of frailty remains. Further studies are required to fully uncover the underlying pathological relationship between frailty and poor prognosis in older HF patients and explore effective intervention procedures to improve frailty and optimize HF prognosis.

## Figures and Tables

**Table 1:** Characteristics of the included studies.

First Author Year Country	N Age (M±SD) Female %	HF Type HFr/pEF % Mean EF %	NYHA Class III-IV %	Follow Up Duration	Frailty Measure	Frailty Prevalence	Frailty Definition
Boxer 2010 USA	59 77.0 28.81	chronic HF HFrEF(100%) NR	42.4	4 years	FP	25.40%	a poor ability to cope with physiologic stress

Cacciatore 2005 Italy	120 75.9± 6.7 60.00	chronic HF NR NR	NR	12 years	FSS	54.2% (FSS 2-3)	includes not only hospitalization, health service utilization, caregiver stress, but also age–associated sensory deficits, functional impairment or degree of disability, cognitive and behavioural deficits, and lack of social support

Martín-Sánchez 2017 Spanin	465 82.4 ±7.2 61.00	Acute HF NR NR	23.1	30days	Modified FP(self reported)	36.30%	a dynamic and nonlinear process that describes a state of vulnerability (reduced system reserves and capacity of response to stress situations) to stressors in older populations

Pulignano 2006 Italy	190 77.0±5.0 46.30	chronic HF NR 32.94±10	NR	1year	FSS	40% FSS(2-4)	a clinical state of increased vulnerability to adverse outcomes such as disability and mortality

Rodríguez-Pascual 2017 Spanin	497 85.2±7.3 61.00	chronic HF HFpEF (79.3%) NR	27.9	1year	FP	57.50%	an age-associated medical syndrome characterised by increased vulnerability to even minor stressors, which manifests as higher risk of adverse health outcomes including disability, hospitalisation and death

Vidán 2016 Spanin	416 80.0±6.1 49.50	chronic HF HFpEF (51.0%) NR	25.5	1 year	FP	76%	a geriatric syndrome of increased vulnerability to stressors due to cumulative declines across different physiological systems

Chaudhry 2013 USA	758 79.7±6.2 50.50	Newly diagnosed HF HFpEF (56.8%) NR	30.3	20 years	Grip strength Gait speed	41.8% (for both grip strength and gait speed)	NR

Chiarantini 2010 Italy	157 80.0±0.5 49.70	Decompensated HF HFpEF (46.0%) 43.4±14.7	59.9	median follow-up of 444 days	SPPB	50.9% (SPPB 0-4)	NR

Madan 2016 USA	40 74.9± 6.5 57.50	Advanced HF HFrEF(100%) 25.6 ± 6.4	100	454 ± 186 days	FP	65%	a biological syndrome defined as a decreased homeostatic reserve leading to an increased vulnerability to stressors and adverse outcomes

Pulignano 2016 Italy	331 78.0±5.2 42.30	chronic HF HFpEF (19.9%) 34.6±11.6	51.4	1year	Gait speed	34.7% (⩽0.65 m/s)	a syndrome of loss of reserves that enhances vulnerability to stressors (e.g., concomitant acute illnesses, hospitalizations, medical procedures)

Abbreviations: EF, Ejection Fraction; FP, Frailty Phenotype; FSS, Frailty Staging System; HF, heart failure; HFpEF, Heart Failure preserved Ejection Fraction; HFrEF, Heart Failure reduced Ejection Fraction; M, mean; N, number; NR, no report; NYHA, New York Heart Association; SD, standard deviation; SPPB, Short Physical Performance Battery; USA, United States of America.

**Table 2:** Main findings and quality of eligible studies.

First Author	Endpoints	Comparisons	Association	Adjusted Factors	NOS
Boxer	mortality	frail/nonfrail	HR 1.58; 95%CI=1.15-2.17 P=0.005	Adjusted for age (5-year categories); CRP levels; NYHA classifications; interleukin-6	9

Cacciatore	mortality	frail class(2-3)/ 1	HR 1.48; 95%CI=1.04-2.11 P=0.032	Adjusted for age; sex; NYHA class; comorbidity; systolic blood pressure; diastolic blood pressure; diuretics; ACE-inhibitors; nitrates and digoxin and ischaemic aetiology	9

Martín-Sánchez	all-cause mortality	frail/nonfrail	HR 2.5; 95% CI = 1.0–6.0; P= 0.047	Adjusted by sex; arterial hypertension; atrial fibrillation; previous diagnostic of heart failure; Barthel index; baseline NYHA class; tachycardia; hypoxemia; anemia; CHF risk model; NT-proBNP	7

Pulignano(2006)	1-year mortality 1-year HF hospital re-admissions	frail class(2-4)/1	HR 1.74; 95% CI= 1.10-2.75 HR 3.11; 95% CI= 1.61-6.03	Advanced age; EF<20%; SBP<100 mmHg; anemia no BB therapy	7

Rodríguez-Pascual	all-cause mortality, readmission incident functional limitation	frail/nonfrail	HR 2.15; 95% CI=1.23–3.76; P=0.005 HR 1.65; 95% CI=1.11–2.46; P<0.05 HR 1.55; 95% CI=0.91–2.66; P<0.05	Adjusted for age, sex, dementia, serum creatinine level, limitation in IADL, NYHA III–IV functional class, Charlson comorbidity index, LVEF ⩽ 45%, previous admission due to HF, treatment with beta-blockers, and treatment with ACEI/ARB.	8

Vidán	1-year all-cause mortality 1-year readmission 30-day functional decline	frail/nonfrail	HR 2.13; 95%CI=1.07–4.23; P=0.031 OR 1.96; 95% CI=1.14–3.34; P<0.05 OR 2.20; 95% CI=1.19–4.08; P<0.05	Adjusted for age; gender; chronic co-morbidity; presence of other acute diseases; LVEF; NYHA class; NT-proBNP levels	9

Chaudhry	all-cause hospitalizations	weak grip/normal slow gait/normal	HR 1.19; 95%CI=1.00–1.42; P=0.050 HR 1.28; 95%CI=1.06–1.55; P=0.010	Adjusted for Demographics (age sex education); Heart failure Status (Ejection fraction < 45% NYHA III/IV Not taking Beta-blocker); Medical history(Diabetes mellitus Chronic kidney disease Stroke); Depression	9

chiarantini	mortality	SPPB0 or 1-4 /SPPB9-12	0 HR6.06; 95% CI=2.19-16.76; P=0.001 1-4HR4.78; 95%CI=1.63-14.02; P=0.004	Adjusted for demographics; study site; left ventricular ejection fraction; comorbidity; New York Heart Association class	8

Madan	all-cause hospitalization or death all-cause hospitalizations subgroup non-HF–related hospitalizations HF-related hospitalizations	frail/prefrail	HR 1.95; 95% CI=1.06–3.59; P =0.031 HR 1.92; 95% CI=1.12–3.27; P =0.017 subgroup HR 3.31; 95% CI=1.14- 9.6; P =0 .028 HR 1.31; 95% CI=0.68–2.49; P = 0.380	Adjusting for diabetes; age; sex	9

Pulignano(2016)	all-cause mortality all cause hospitalization HF-related hospitalizations	highest tertiles/the lowest tertile	HR 0.62; 95% CI= 0.43 -0.88; P=0.008 HR 0.74; 95% CI= 0.61 -0.90; P=0.002 HR 0.70; 95% CI= 0.55 -0.90; P=0.004	Adjusted by Age; SBP; No beta-blocker therapy; NYHA class III/IV (yes vs. no); LVEF <20%; Anemia	8

Abbreviations: ACE, angiotensin-converting enzyme; ACEI, angiotensin converting enzyme inhibitors; ARB, angiotensin receptor blockers; CHF, congestive heart failure; CI, confidence interval; CRP, C-reactive protein; HR, hazard ratio; IADL, Instrumental Activities of Daily Living; LVEF, left ventricle ejection fraction; NT-proBNP, N-terminal prohormone B-type natriuretic peptide; NYHA, New York Heart Association; SBP, systolic blood pressure.
